# Sleep Disturbances and Sleep Disordered Breathing Impair Cognitive Performance in Parkinson’s Disease

**DOI:** 10.3389/fnins.2020.00689

**Published:** 2020-08-06

**Authors:** Wiebke Hermann, Henning Schmitz-Peiffer, Elisabeth Kasper, Mareike Fauser, Christiana Franke, Miriam Wienecke, Karolin Otto, Matthias Löhle, Moritz D. Brandt, Heinz Reichmann, Alexander Storch

**Affiliations:** ^1^Department of Neurology, Technische Universität Dresden, Dresden, Germany; ^2^Sleep Centre, Department of Neurology and Department of Internal Medicine, Technische Universität Dresden, Dresden, Germany; ^3^Department of Neurology, University of Rostock, Rostock, Germany; ^4^German Center for Neurodegenerative Diseases (DZNE), Research Site Rostock, Rostock, Germany; ^5^German Center for Neurodegenerative Diseases (DZNE), Research Site Dresden, Dresden, Germany

**Keywords:** Parkinson’s disease, sleep disorders, sleep disordered breathing, sleep apnea, cognition, cognitive performance

## Abstract

**Background:**

Sleep disturbances and impairment of cognitive function are among the most frequent non-motor symptoms in Parkinson’s disease (PD) with negative implications on quality of life of patients and caregivers. Despite the fact that sleep disturbances are a major issue in PD patients, only limited data are available regarding interactions of sleep disturbances and cognitive performance.

**Objective:**

This *post hoc* analysis of the RaSPar trial was therefore designed to further elucidate sleep disturbances and their impact on cognition in PD.

**Methods:**

Twenty-six PD patients with sleep disturbances were evaluated thoroughly including assessments of patients’ subjective and objective sleep quality by interview, questionnaires, and polysomnography (PSG). Cognitive performance was assessed by Parkinson Neuropsychometric Dementia Assessment (PANDA) and Test of Attentional Performance (TAP), and associations of sleep and cognitive function were evaluated.

**Results:**

We did not detect differences in cognitive performance between patients with and without rapid eye movement (REM) sleep behavior disorder (RBD). Instead, cognitive impairment, particularly affecting cognitive domains attention, executive function/working memory, and semantic memory, was associated with impaired PSG-measured sleep quality (e.g., sleep efficiency) and sleep disordered breathing (SDB) (Apnea-Hypopnea Index > 5/h). Global cognitive performance was decreased in patients with SDB (PANDA score 23.2 ± 3.5 vs. 26.9 ± 2.2, *P* = 0.020, unpaired two-sided *t*-test).

**Conclusion:**

Sleep apnea and other sleep disturbances impair cognitive performance in PD and should be evaluated in routine care, and treatment options such as continuous airway pressure therapy should be considered.

## Introduction

Parkinson’s disease (PD) is one of the most common age-related neurodegenerative disorders primarily defined by motor symptoms such as tremor and akinesia. The importance of non-motor symptoms like sleep, autonomic, cognitive, or psychiatric disturbances on functioning and quality of life of patients and caregivers has been more and more recognized over the past few years (Dhawan et al., 2006; De Cock et al., 2008; Barone et al., 2009; Chaudhuri et al., 2011; Martinez-Martin et al., 2011; Hirata et al., 2015; Hiseman and Fackrell, 2017). Sleep disorders are among the most frequent and important non-motor symptoms in PD affecting up to 90% of patients. However, these symptoms are still underreported by patients and underdiagnosed by health care professionals (Tandberg et al., 1998; Dhawan et al., 2006; Alatriste-Booth et al., 2015; Albers et al., 2017). Sleep disturbances in PD include excessive daytime sleepiness (EDS), insomnia, circadian rhythm disorders, restless-legs syndrome and periodic limb movements in sleep (PLMS), sleep disordered breathing (SDB) such as obstructive sleep apnea (OSA), as well as parasomnias like rapid eye movement (REM) sleep behavior disorder (RBD). Reasons for sleep disturbances in PD are multifaceted comprising neurodegeneration especially in the brain stem, thalamus, hypothalamus, and cortical areas affecting sleep wake regulation mechanisms and neurotransmitters complemented by negative influences of nighttime motor impairment, non-motor symptoms like nocturia, and adverse effects of treatment regimens, as well as concomitant primary sleep disorders such as SDB.

Cognitive impairment in PD reaches from mild subjective and mild objective decline to severe dementia [Parkinson’s disease dementia (PDD)] with an elevated prevalence of about 25–40% in cross-sectional studies compared to the general population (Aarsland et al., 2010, 2017). In the long-term follow-up, 15–20 years after disease onset, up to 80% of PD patients may develop cognitive impairment (Hely et al., 2008; Aarsland and Kurz, 2010; Jozwiak et al., 2017). The risk for dementia in PD increases with disease progression and seems to be associated with the presence of RBD (Jozwiak et al., 2017), akinetic PD subtype (Svenningsson et al., 2012), age, and orthostatic hypotension (Anang et al., 2014). Cognitive deficits in PD patients are diverse with particular impairment in attentional, executive function, episodic learning, memory, and visuospatial domains (Dubois et al., 2007; Aarsland et al., 2011). Several mechanisms including spread of α-synuclein Lewy pathology to limbic and neocortical structures (Braak et al., 2005), involvement of basal ganglia and the pedunculopontine nucleus (PPT) (Targa et al., 2018), and hyperphosphorylated tau- as well as amyloid-β deposition (Hepp et al., 2016) seem to contribute to cognitive decline in PD. However, in comparison to Alzheimer’s disease, memory impairment in PD seems to result predominantly from ineffective strategies in encoding and retrieval due to executive dysfunction (Pillon et al., 1993; Bosboom et al., 2004; Kalbe et al., 2008).

In the general population, healthy sleep in young adults is associated with memory consolidation and synaptic plasticity (Feld and Born, 2017). On the contrary, a bidirectional relation of sleep and cognitive performance was suggested in recent publications with sleep disturbances being associated with aggravation of cognitive impairment and an increased risk to develop dementia (Scullin and Bliwise, 2015; Shi et al., 2018). Accordingly, in a large cohort study including 197 PD patients, sleep disturbances were associated with impaired visuospatial functioning and visual learning and memory (Specketer et al., 2019). Although recent evidence strongly suggests a link between sleep and cognitive performance in other sleep disorders such as insomnia and SDB (Yaffe et al., 2011; Osorio et al., 2015), the association of cognitive performance and sleep in PD has only been evaluated to a limited extent—particularly with regard to polysomnographic (PSG) sleep evaluation—compared to studies only using actigraphy or questionnaires (Stavitsky et al., 2012; Goldman et al., 2013; Scullin et al., 2015; Specketer et al., 2019). Furthermore, the influence of SDB on cognition in PD is ambiguous with some authors arguing against an association in PD (Beland et al., 2015; Scullin et al., 2015). In contrast, others showed impaired global cognitive performance in PD patients with sleep apnea (Mery et al., 2017) with attention, executive function, and visuospatial abilities as the most affected domains (Neikrug et al., 2013; Harmell et al., 2016). Likewise, nocturnal oxygen desaturation was shown to impair cognitive performance, particularly attention and executive function, in patients with mild cognitive impairment (MCI) and dementia (Yamout et al., 2012). Accordingly, continuous positive airway pressure (CPAP) treatment was able to improve global cognitive performance in PD patients diagnosed with moderate to severe SDB (Kaminska et al., 2018). However, other authors reported significant adherence problems and were not able to detect improvement of cognition with CPAP treatment (Terzaghi et al., 2017).

Identification of PD patients at risk of rapid cognitive decline is important for prognosis and patient management, and sleep disturbances might be a treatable cause for cognitive decline. Therefore, the current *post hoc* analysis was performed to further evaluate the association of sleep disturbances and cognitive performance, especially attention, in PD patients by comprehensive evaluation of cognitive measures and subjective and objective sleep evaluation by questionnaires and PSG.

## Subjects and Methods

### Study Participants

Patients with PD according to the UK Parkinson’s Disease Society Brain Bank clinical criteria (Hughes et al., 1992) aged 50–80 years with a modified Hoehn and Yahr stage between 1 and 3 (Hoehn and Yahr, 1967) were screened for sleep disturbances using the Pittsburgh Sleep Quality Index (PSQI), a self-rating questionnaire to assess global sleep quality (scores >5 indicating disturbed sleep, range 0–21) (Buysse et al., 1989; Pal et al., 2004) at the Movement Disorders Center of the Department of Neurology of the Technische Universität Dresden. If sleep disturbances were suspected based on the PSQI screening, sleep quality was further evaluated by a semi-standardized interview of patients and caregivers—if available—as well as sleep questionnaires and PSG to obtain objective sleep parameters.

From 29 patients initially enrolled between 2011 and 2014, two were excluded due to cognitive impairment [Parkinson Neuropsychometric Dementia Assessment (PANDA) score <18] and one due to diagnostic precariousness, thus, 26 PD patients were included in the analysis. Patients fulfilling PSG exclusion criteria {e.g., severe SDB [Apnea-Hypopnea Index (AHI) >30/h]} and patients with signs of atypical parkinsonian syndromes, deep brain stimulation, severe depression [Montgomery–Asberg Depression Scale (MADRS) >34], severe dementia, or other medical conditions interfering with study procedures as well as patients on medication influencing sleep, e.g., hypnotics and antidepressants, were excluded from the study [for details, please refer to Schrempf et al. (2018)]. Patients had to be on stable antiparkinsonian medication for at least 4 weeks prior to study inclusion.

### Clinical Assessments

After signed informed consent was received, participants meeting study inclusion criteria were assessed using Unified Parkinson’s Disease Rating Scale [UPDRS, part I motivation, part II activities of daily living (ADL), part III motor impairment, part IV complications] (Martinez-Martin et al., 1994), Schwab and England (S&E) to assess activities of daily living, MADRS to evaluate depressive symptoms (Schmidtke et al., 1988), and Parkinson’s Disease Quality of Life Questionnaire (PDQ-39) to assess quality of life (Jenkinson et al., 1995). Subjective sleep disturbances were thoroughly evaluated using a semi-standardized interview comprising questions to assess symptoms of sleep onset insomnia (SOI) and sleep maintenance (SM) insomnia (SMI), EDS, SDB, restless-legs symptoms, and dream enactment behavior. Furthermore, subjective sleep quality was evaluated by self-rating questionnaires (PSQI (Buysse et al., 1989; Pal et al., 2004), Parkinson’s Disease Sleep Scale-2 [(PDSS-2) (Trenkwalder et al., 2011), a self-rating, sleep quality questionnaire especially addressing sleep disturbances in PD (range 0–60, scores >18 are considered relevant sleep disturbances (Muntean et al., 2016)], and Epworth Sleepiness Scale [(ESS) range 0–24 points, scores >10 suggesting relevant daytime sleepiness (Johns, 1991)]). Cognitive performance was evaluated using an overall screening tool [PANDA (Kalbe et al., 2008)] to assess modd/depressive symptoms and different cognitive domains (memory, executive function including working memory/verbal fluency, attention, visuospatial abilities) with a total score of 30 resulting by transformation of raw data into normalized values. Within the PANDA, the word pair associate learning task with immediate and delayed recall has been established to test memory, but also includes executive function and attention. The alternating semantic verbal fluency task was designed to assess working memory (executive function) and semantic memory, but also assesses other domains such as attention and processing speed. The visuospatial task was designed to measure visuospatial function, and digit spans were designed to assess working memory. The Test of Attentional Performance (TAP) (Catale et al., 2009) with the subtests alertness, divided attention, and response inhibition (Go/No-Go paradigm) was used to further evaluate the cognitive domains attention/divided attention, alertness, executive function/response inhibition, and processing speed. Due to technical issues, cognitive testing was performed within 2 weeks before PSG recording, thus preventing a negative influence of the PSG itself on cognitive performance.

We used the following defined clinically important cutoffs to define possible MCI (PANDA scores: 15–17 points) and possible dementia (PANDA scores: <15). We classified sleep disorders according to International Classification of Sleep Disorders-Third Edition (ICSD-3) (American Academy of Sleep Medicine (AASM), 2014a). All clinical ratings were assessed by movement disorder trained physicians blinded to the PSG data (MW, ML, MDB, MF, CF, and WH). Levodopa equivalent doses were calculated according to Tomlinson et al. (2010). The presence of specific sleep complaints such as SMI, SOI, or EDS was based on the semi-standardized interviews. Additionally, EDS was classified based on the questionnaire if the sum of the ESS score was >10 (Johns, 1991). Disease duration was defined as the time since PD diagnosis. We report here the detailed baseline characteristics of the study cohort without study-related interventions (Schrempf et al., 2018).

### Polysomnographic Assessment

After the screening visit, patients found eligible underwent full-night attended, digital video-PSG (polysomnography) measurement [Alice 5.0 software, Löwenstein Medical GmbH, Germany] at the Dresden University Sleep Centre comprising of electroencephalography (EEG), electrooculogramm (EOG), and electromyography (EMG) of the Musculus submentalis and both Mm. anterior tibialis, oronasal airflow, microphone, thoracic and abdominal respiratory effort, position sensor, oxygen saturation, and electrocardiogram (ECG) according to AASM standard recommendations (American Academy of Sleep Medicine (AASM), 2014b). PSG was scored manually and evaluated by a trained certified sleep specialist (WH). PSG-based objective sleep measurements included sleep efficiency [SE = total sleep time (TST)/time in bed (TIB, time from lights off to lights on)] and SM [SM = TST/sleep period time (SPT, time from first epoch of sleep to lights on)], frequency of sleep stages N1, N2, N3 and REM, sleep latency, REM latency, TST, wake time, Arousal Index (AI), PLMS, AHI, Respiratory Distress Index (RDI), and Oxygen Desaturation Index (ODI). The presence of RBD was diagnosed according to actual standard recommendations of the AASM and using SINBAR PSG scoring criteria (Frauscher et al., 2012; American Academy of Sleep Medicine (AASM), 2014b).

### Statistical Analysis

Baseline characteristics of the study population were analyzed by mean [standard deviation (SD)] or median [interquartile range (IQR)]. Correlations were performed using Pearson’s correlation test or Spearman rank correlation test for continuous and ordinal variables as appropriate and χ^2^ test for nominal variables. Subgroups of patients were analyzed, e.g., patients with and without the presence of SDB (AHI >5/h vs. AHI ≤5/h). Furthermore, patients were grouped according to the presence of RBD as RBD-positive (RBD+) and RBD-negative (RBD-) subgroup based on PSG evaluation. Between-group analyses were performed using unpaired two-sided Student’s *t*-test (parametric continuous variables) or Mann–Whitney *U* test (non-parametric continuous variables) or chi-square test/Fisher’s exact test (discrete variables) as appropriate. Pearson’s correlation test and multivariate linear regression modeling with a stepwise approach was used for correlations of cognitive measures and candidate covariates potentially influencing cognitive performance such as age, sex, PD symptom duration and disease severity (UPDRS III motor score), presence of RBD, SE, and AHI. κ or Pearson’s correlation coefficient |*r*| < 0.3 was considered a weak, κ/|*r*| = 0.3–0.59 a moderate, and κ/|*r*| ≥ 0.6 a strong agreement/correlation. All reported *P*-values are two-sided. If not mentioned otherwise, all results are presented as mean values ± SD, median (IQR), numbers (n), or percentages (%); the significance level was set at *P* < 0.05 (two-tailed test). Due to the explorative character of the study, α adjusting of *P*-values (e.g., Bonferroni method) for multiple testing was not performed. Statistical analyses were performed using IBM SPSS version 23.0 or higher (IBM Corporation, Armonk, NY, United States).

## Results

### Study Participants

26 PD patients were included into the analysis [age 70.9 ± 6.2 years; 15 male; Hoehn and Yahr (H&Y) 2.0 ± 0.8] (Table 1). Evaluation of subjective sleep complaints as assessed by the semi-standardized interview showed SMI as the most frequent sleep disturbance reported by 93% of PD patients, followed by EDS (89%). Other sleep complaints comprised snoring (73%), SOI (35%), and features of RBD such as dream enactment behavior (48%) and talking or shouting during sleep (64%).

**TABLE 1:** Clinical characteristics of total cohort.

	**Total cohort (*n* = 26)**
**Demographics and clinical characteristics**	
Age (years), mean ± *SD*	70.9 ± 6.2 (56–80)
Men/women (*n*)	15 m; 11 f
Education (overall, years)	14.4 ± 2.9 (10–20)
Education (school, years)	10.5 ± 1.8 (8–12)
Education ≤ 10 years	1/26 (38%)
Education ≤ 13 years	9/26 (35%)
Education > 13 years	12/26 (46%)
PD symptom duration (years), mean ± *SD*	3.9 ± 3.7 (0–15)
PD duration (years), mean ± *SD*	2.5 ± 2.9 (0–10)
UPDRS total score	32.3 ± 11.3 (10–60)
UPDRS part I (psychiatric)	2.6 ± 1.4
UPDRS part II	8.3 ± 3.7
UPDRS part III (motor function)	18.9 ± 7.7
UPDRS part IV (motor complications)	2.6 ± 1.9
Modified Hoehn and Yahr stage	2.0 ± 0.8
1	7 (27%)
2	8 (31%)
2.5	5 (19%)
3	6 (23%)
Schwab and England Scale ADL	86 ± 8.7 [25]
PDQ-39 sum score	39.4 ± 27.5 [25]
PDSS-2	19.3 ± 8.7 [25]
ESS	8.6 ± 5.0 [25]
ESS < 10	17/25 (68%)
ESS > 10	8/25 (32%)
PSQI	9.7 ± 2.8
MADRS	9.4 ± 6.4
PANDA (mood)	3 ± 2.3 [23]
PANDA (cognition)	24.6 ± 3.6 [24]
PANDA ≤ 14	0/24 (0%)
PANDA 15–17	0/24 (0%)
PANDA ≥ 18	24/24 (100%)
BMI (kg/m^2^KOF)	26.9 ± 3.9 (18–35)
Blood pressure systolic (mmHg)	138.9 ± 23 (95–195)
Blood pressure diastolic (mmHg)	79.4 ± 10.9 (56–102)
History of RBD (violent behavior)	12/25 (48%)
History of talking in sleep	16/25 (64%)
Polysomnographically proven RBD	17/25 (68%)
**PD subtype**	
Akinetic type	9/26 (35%)
Tremor dominant type	7/27 (27%)
Equivalent type	10/27 (39%)
**Antiparkinsonian medication**	
Levodopa	15/26 (58%)
Dopamine agonists	15/26 (58%)
COMT inhibitors	1/26 (4%)
Amantadine	0/26 (0%)
*De novo* PD patients	4/26 (15%)
Levodopa equivalent dose (mg/day), (mean ± *SD*, range)	301.3 ± 234 (0–860)

*Data are mean (±SD) or numbers [n]/percentage (%). ADL, activities of daily living; BMI, body mass index; COMT, catechol-O-methyltransferase; ESS, Epworth sleepiness scale; LED, levodopa equivalent dose; MADRS, Montgomery–Asberg Depression Rating Scale; PANDA, Parkinson Neuropsychometric Dementia Assessment; PD, Parkinson’s disease; PDQ-39, Parkinson’s Disease Questionnaire; PDSS-2, Parkinson’s Disease Sleepiness Scale 2; PSQI, Pittsburgh Sleep Quality Index; UPDRS, Unified Parkinson’s Disease Rating Scale (part I: evaluation of mentation, behavior and mood; part II: activities of daily life; part III: motor function; part IV: complications). ^*a*^Levodopa equivalent dose was calculated according to Tomlinson et al. (2010).*

### Polysomnographic Sleep Measures

Polysomnography recordings demonstrated reduced SE, SM, TST, and percentage of REM sleep. Furthermore, increased REM latency, wake time in TIB, and PLM Index, as well as fragmentation of sleep structure with an elevated AI—as already known in PD patients—were detected (Supplementary Table S1). The mean AHI was slightly elevated (10.9 ± 8.5/h) compared to normal values (cutoff AHI ≤ 5/h). SDB (cutoff AHI > 5/h) was detected in 73% of patients (Supplementary Table S1) with 46% (12/26) of our patients classified as suffering from mild to moderate sleep-related breathing disorder (SDB, AHI > 5/h ≤ 15/h), whereas 27% (7/26) were found to have moderate SDB (AHI > 15/h ≤ 30/h). Patients with severe SDB (AHI > 30/h) were not recruited into the study according to the predefined exclusion criteria (Schrempf et al., 2018).

### Cognitive Measures

The total score of the cognitive part of the PANDA was normal in all PD patients (24.6 ± 3.6; Table 1) according to exclusion criteria. In contrast, when analyzing cognitive performance using normative *t*- and *z*-scores, impaired performance in all TAP tasks with even more prominent deficits in the alertness and divided attention tasks—particularly divided attention with auditory cue—compared to a more preserved response inhibition were detectable (Table 2).

**TABLE 2:** Cognitive performance of the overall cohort.

		**Total cohort**	**Max. possible scores**
**PANDA (mean ± *SD*) [*n* = 24]**			
PANDA (cognition) – overall result		24.3 ± 3.6 (range 18–30)	
**Domains memory/executive function**			
Word pair associate learning – immediate recall (raw score)		7.4 ± 2.5	12
Word pair associate learning – immediate recall (transformed)		4.3 ± 0.9	5
Word pair associate learning – delayed recall (raw score)		2.5 ± 1.1	4
Word pair associate learning – delayed recall (transformed)		5.3 ± 1.5	7
**Domains executive function (working memory)/semantic memory/attention/processing speed**			
Alternating verbal fluency (raw score)		13.1 ± 3.3	–
Alternating verbal fluency (transformed)		5.7 ± 1.5	7
**Domain visual perception (max. score 3)**			
Visuospatial task (raw score)		2.4 ± 0.8	3
Visuospatial task (transformed)		4.2 ± 1.2	5
**Domains executive function/attention (max. score 6)**			
Working memory (raw score)		5.3 ± 0.7	–
Working memory (transformed)		5.1 ± 1.1	6
Attention task (errors) (raw score)		0.8 ± 1.9	–

**TAP (mean ± *SD*) [*n* = 14]**	**Total cohort**	**Normative *t*-values**	**Normative *z*-values**

**Domains alertness/processing speed**			
Alertness task without auditory cue (median) [ms] [14]	302.6 ± 54.2 [294.5 (253–337)]	40.9 ± 8.9 [40 (33–49)]	−0.9 ± 0.9 [−1.0 (−1.7–−0.8)]
Alertness task with auditory cue (median) [ms] [14]	295.5 ± 57 [284.5 (251–336)]	39.3 ± 7.9 [39 (33–45)]	−1.1 ± 0.8 [−1.1 (−1.7–−0.5)]
**Domains executive function (response inhibition)/attention**			
Response inhibition Go/No-Go task (median) [ms] [13]	475.1 ± 70.4 [466 (410–518)]	45.4 ± 9.7 [46 (38–54)]	−0.5 ± 1.0 [−0.4 (−1.2–0.4)]
Response inhibition (Go/No-Go) task (errors) [*n*] [13]	1.9 ± 1.7 [2 (0.5–2.5)]	48.5 ± 11.4 [44 (42–59)]	−0.2 ± 1.1 [−0.6 (−0.8–0.9)]
**Domains attention/executive function**			
Divided attention (auditory task) (median) [ms] [13]	734.6 ± 227.5 [687 (601–843)]	37 ± 14.9 [36 (25–46)]	−1.3 ± 1.5 [−1.4 (−2.6–0.4)]
Divided attention (visual task) (median) [ms] [13]	965.7 ± 173.2 [839–1,104)]	44.4 ± 11.5 [47 (36–52)]	−0.6 ± 1.2 [−0.3 (−1.5–0.2)]
Divided attention (errors) (*n*) [13]	4.7 ± 5.1 [2 (1–7.5)]	42.1 ± 10.2 [44 (33–52)]	−0.8 ± 1.0 [−0.6 (−1.7–0.2)]
Divided attention (missed) (*n*) [13]	5.8 ± 5.1 [13] [4 (2–11.5)]	39.3 ± 10 [39 (29–48)]	−1.1 ± 1.0 [−1.1 (−2.2–−0.2)]

*Baseline values of cognitive measures (raw scores and transformed scores) of PANDA as well as TAP. Data are presented as means ± SD, numbers [n], and [median (IQR)]. PANDA, Parkinson Neuropsychometric Dementia Analysis; TAP, Test for Attentional Performance.*

Subjective sleep disturbances—as assessed by the semi-standardized interview—were associated with cognitive performance, predominantly attention and executive function, but also with memory. In detail, the presence of subjective EDS was associated with impaired memory, executive function, response inhibition, and attention, as measured by the PANDA word pair task and number of errors in the Go/No-Go task of the TAP (*P* = 0.042 and *P* = 0.023, respectively; both χ^2^ test). Patients with SOI showed attentional deficits at the TAP divided attention visual task compared to patients without (1,241.5 ± 122.3 ms vs. 915.6 ± 128.6 ms, *P* = 0.007, unpaired two-sided *t*-test). However, subjective impairment of SM was associated with decreased performance in the verbal fluency task of the PANDA, indicating not only attentional deficits but also executive dysfunction (working memory), as well as impaired semantic memory and processing speed, compared to patients without SMI [12.5 ± 3.1 vs. 17 ± 2.6 (raw score), *P* = 0.011; Mann–Whitney *U* test]. Verbal fluency was also hindered in patients whose caregivers or spouses were aware of and reported sleep apneas [11 ± 0 vs. 13.4 ± 3.5 (raw score), *P* = 0.011; Mann–Whitney *U* test]. History of leg movements in sleep (PLMS), possibly influencing sleep continuity, was associated with increased error rates in the working memory/executive function task of the PANDA (*P* = 0.031; χ^2^ test).

Overall performance in the cognitive part of the PANDA was negatively associated with age (|*r|* = −0.443, *P* = 0.030 Pearson correlation test; Table 3 and Supplementary Table S2). Furthermore, disease severity measured by the H & Y stage and the UPDRS part IV assessing motor complications were associated with impairment of executive function/response inhibition (|*r*| = 0.668, *P* = 0.013 and |*r|* = 0.643, *P* = 0.018, respectively, Spearman correlation test). The PSQI as a global measure of subjective sleep disturbances was negatively associated with executive function, attention, processing speed, and memory, as assessed by the PANDA word pair associations, attention, and verbal fluency tasks (Supplementary Table S2). Cognitive measures were not associated with any other clinical data, questionnaires, or RBD status.

Polysomnography-derived objective measures of sleep quality such as SE, SM, and TST, were negatively associated with attention (Supplementary Table S2). In detail, SE, SM, and TST were inversely associated with reaction time latencies in the divided attention task of the TAP (SE: |*r*| = −0.640, *P* = 0.018; SM: |*r*| = −0.585, *P* = 0.036; TST: |*r*| = −0.739, *P* = 0.004, respectively, all from Pearson correlation test; Table 3 and Supplementary Table S2). SM was also associated with an increased rate of errors in the attentional task of the PANDA (Supplementary Table S2). REM sleep percentage as a measure of sleep quality and structure was associated with attention, impaired working and semantic memory [PANDA delayed recall of word pairs and TAP divided attention tasks (|*r*| = −0.612 *P* = 0.026 and |*r*| = −0.571 *P* = 0.042, respectively, Pearson correlation test; Supplementary Table S2)]. Additionally, also alertness was affected by impaired sleep quality as indicated by an association with the AI (|*r*| = 0.548, *P* = 0.043, Pearson correlation test).

**TABLE 3:** Multiple linear regression analysis of influencing factors on cognitive performance in PD.

	**Mann–Whitney test**		**Pearson correlation test^§^**					**Multiple linear regression**	
	**Sex**	**RBD (PSG)**	**Age**	**UPDRS III**	**Sleep efficiency**	**AHI (n/h)**	**Education (years)**	**Regression**	**Significant factors**
**PANDA total score**	*P* = 0.796	*P* = 0.256	***|r|* = −0.443**, ***P* = 0.030**	*|r|* = 0.104, *P* = 0.627	*|r|* = 0.255, *P* = 0.229	***|r|*** = −**0.558 *P* = 0.003**	*|r|* = 0.183, *P* = 0.439	***R*^2^ = 0.418, *F* = 12.97, *P* = 0.003**	**AHI: β** = −**0.646, *P* = 0.003**
**PANDA word pair immediate**	*P* = 0.752	*P* = 0.155	***|r|*** = −**0.424 *P* = 0.039**	*|r|* = 0.037, *P* = 0.864	*|r|* = 0.227, *P* = 0.286	*|r|* = −0.053, *P* = 0.406	*|r|* = −0.182, *P* = 0.444	***R*^2^ = 0.267, *F* = 6.182, *P* = 0.024**	**Age: β** = −**0.516, *P* = 0.024**
**TAP-Alertness w/o auditory cue**	*P* = 0.188	*P* = 0.518	*|r|* = −0.499, *P* = 0.070	*|r|* = 0.043, *P* = 0.885	*|r|* = −0.282, *P* = 0.328	*|r|* = 0.099, *P* = 0.369	*|r|* = −0.375, *P* = 0.206	n.s.	
**TAP-Alertness with auditory cue**	*P* = 0.539	*P* = 0.147	*|r|* = −0.378 *P* = 0.183	*|r|* = 0.315, *P* = 0.273	*|r|* = −0.153, *P* = 0.600	*|r|* = −0.010, *P* = 0.486	*|r|* = −0.360, *P* = 0.228	n.s.	
**TAP–Go/No-Go**	*P* = 0.112	*P* = 0.435	*|r|* = −0.200, *P* = 0.512	*|r|* = −0.149, *P* = 0.627	*|r|* = −0.133, *P* = 0.665	*|r|* = 0.235, *P* = 0.220	*|r|* = −0.307, *P* = 0.332	n.s.	
**TAP–divided attention-auditory**	*P* = 0.937	*P* = 0.724	*|r|* = 0.234, *P* = 0.442	*|r|* = −0.104, *P* = 0.736	*|r|* = 0.121, *P* = 0.695	***|r|* = 0.546, *P* = 0.027**	*|r|* = 0.120, *P* = 0.710	n.s.	
**TAP–divided attention-visual**	*P* = 0.112	*P* = 0.724	*|r|* = −0.255, *P* = 0.400	*|r|* = 0.327, *P* = 0.276	***|r|*** = −**0.640, *P* = 0.018**	*|r|* = 0.093, *P* = 0.381	*|r|* = −0.471, *P* = 0.122	***R*^2^ = 0.444, *F* = 7.978, *P* = 0.018**	**Sleep efficiency β** = −**0.666, *P* = 0.018**
**TAP–divided attention (missed, [*n*])**	*P* = 0.287	*P* = 0.622	*|r|* = 377, *P* = 0.204	*|r|* = −0.208, *P* = 0.495	*|r|* = −0.209, *P* = 0.493	***|r|*** = −**0.653, *P* = 0.016**	*|r|* = −0.552, *P* = 0.063	***R*^2^ = 0.442**, ***F* = 7.922**, ***P* = 0.018**	**AHI: β** = −**0.665, *P* = 0.018**

*Cognitive tests are displayed as raw scores using either total score or median ([ms], TAP) if not stated otherwise. ^§^Test results are from multivariate regression analyses with entering the candidate independent variables sex, age, RBD status (PSG), UPDRS III as measure of PD severity, AHI as measure of SBD severity, sleep efficiency. Non-significant p-values of model coefficients have been omitted for clarity. AHI, Apnea-Hypopnea Index; ODI, Oxygen-Desaturation-Index; RBD, REM Sleep Behavior Disorder; PANDA, Parkinson Neuropsychometric Dementia Assessment; PD, Parkinson’s disease; TAP, Test for Attentional Performance; UPDRS, Unified Parkinson’s Disease Rating Scale (part I: evaluation of mentation, behavior and mood; part II: activities of daily life; part III: motor function; part IV: complications). Significant results were marked by bold values.*

However, cognitive performance was also associated with PSG parameters indicating SDB. Global cognitive performance as assessed by the PANDA was moderately associated with severity of SDB, as classified by the AHI (|*r*| = −0.557, *P* = 0.005, Pearson correlation test), RDI, and ODI (Table 3 and Supplementary Table S3). Detailed task-specific analysis revealed influences of SDB on cognitive domains attention, divided attention, executive function/working memory, memory, and to a smaller extent also alertness. In detail, TAP divided attention tasks were strongly correlated with respiratory PSG measures such as the RDI and the RDI in NREM sleep (|*r*| = 0.714, *P* = 0.006; |*r*| = 0.709, *P* = 0.007, Spearman correlation test, respectively) and the AHI (|*r*| = 0.558, *P* = 0.048, Spearman correlation test, Supplementary Table S3). Likewise, PANDA subtests word pairs were associated with SDB-related PSG metrics such as oxygen saturation (SpO_2_) in wake and Non-REM (NREM) sleep (Supplementary Table S3).

### Sleep Disordered Breathing

When we analyzed subcohorts of PD patients with SDB (SDB+, AHI > 5/h, *n* = 19) and without SDB (SDB−, AHI ≤ 5/h, *n* = 7), demographic and clinical characteristics including body mass index (BMI) did not differ significantly apart from blood pressure values, which were significantly higher in patients with SDB (Table 4). Obviously, SDB− patients had less respiratory-related events such as AHI (1.8 ± 1.5 vs. 14.2 ± 7.4, *P* < 0.001) and ODI (0.6 ± 0.9 vs. 8.3 ± 5.7, *P* < 0.001, all unpaired two-sided *t*-test), whereas PSG measures of sleep quality such as SE did not differ between patients with and without SDB (Supplementary Table S4).

**TABLE 4:** Clinical characteristics of patient subcohorts with sleep disordered breathing (SDB, AHI > 5/h) and without SDB (AHI ≤ 5/h).

	**SDB+ AHI > 5/h**	**SDB− AHI ≤ 5/h**	***P*-value**
	***n* = 19**	***n* = 7**	
Age (years), mean ± *SD*	71.4 ± 6.5 (56–80)	69.6 ± 5.6 (60–76)	0.364^‡^
Men/women (*n*)	12 m; 7 w	3 m, 4 f	0.407^‡‡‡^
PD symptom duration (years), mean ± *SD*	4.0 ± 4.2 (0–15)	3.6 ± 1.9 (1–6)	0.692^‡^
PD duration (years), mean ± *SD*	2.4 ± 3.2 (0–10)	2.9 ± 2.0 (1–6)	0.231^‡^
UPDRS total score	33.6 ± 12.7	28.9 ± 5.8	0.357^§^
UPDRS part I (psychiatric)	2.7 ± 1.3	2.1 ± 1.6	0.395^‡^
UPDRS part II	8.6 ± 4.2	7.6 ± 1.3	0.545^§^
UPDRS part III (motor function)	19.6 ± 8.3	16.7 ± 5.7	0.404^‡^
UPDRS part IV (motor complications)	2.6 ± 2.0	2.4 ± 1.6	0.910^‡^
Modified Hoehn and Yahr stage	2.1 ± 0.8	1.9 ± 0.7	0.572^‡^
1	5/19 (26%)	2/7 (29%)	
2	5/19 (26%)	3/7 (43%)	
2.5	4/19 (21%)	1/7 (14%)	
3	5/19 (26%)	1/7 (14%)	
Schwab and England Scale ADL	85.3 ± 9.6 (70–100)	88.3 ± 4.1 (80–90)	0.475^‡^
PDQ-39 sum score	39.4 ± 28.8	39.3 ± 26.2	0.990^§^
PDSS-2	17.8 ± 7.7	23.1 ± 10.5	0.175^§^
ESS	8.6 ± 5.2 [18]	8.7 ± 4.8	0.964^§^
PSQI	9.7 ± 2.6	9.7 ± 3.4	0.986^§^
MADRS	10.6 ± 6.5	6.1 ± 5.1	0.063^‡^
PANDA (mood) – result	3.3 ± 2.4	2.3 ± 2.0	0.384^§^
BMI	27.5 ± 3.4 (22–35)	25.3 ± 5.0 (18–33)	0.214^§^
BP syst.	146.6 ± 21.3 (108–195)	118 ± 11.6 (95–132)	0.003⁢**§
BP diast.	82.8 ± 10.1 (66–102)	70.1 ± 7.1 (56–76)	0.006⁢**§
**Antiparkinsonian medication**			
Levodopa	11/19 (58%)	4/7 (57%)	1.000^‡‡‡^
Dopamine agonists	11/19 (58%)	4/7 (57%)	1.000^‡‡‡^
COMT inhibitors	1/19 (5%)	0 (0%)	1.000^‡‡‡^
Amantadine	0 (0%)	0 (0%)	
^a^Levodopa equivalent dose (mg/day), (mean ± *SD*, range)	298.3 ± 248.5 (0–860)	309.5 ± 206.9 (0–620)	0.916^§^

Data are mean ± SD, numbers [n], percentages (%) or range (min-max) as appropriate. P-values are from ^§^Student’s t-test, ^‡‡‡^Fisher’s exact test or ^‡^Mann–Whitney U test as appropriate. **p < 0.01. ADL, activities of daily living; BMI, body mass index; BP, blood pressure; COMT, Catechol-O-methyltransferase; ESS, Epworth sleepiness scale; LED, levodopa equivalent dose; MADRS, Montgomery–Asberg Depression Rating Scale; PANDA, Parkinson Neuropsychometric Dementia Assessment; PD, Parkinson’s disease; PDQ-39, Parkinson’s Disease Questionnaire; PDSS-2, Parkinson’s Disease Sleepiness Scale 2; PSQI, Pittsburgh Sleep Quality Index; UPDRS, Unified Parkinson’s disease rating scale (part I: evaluation of mentation, behavior, and mood; part II: activities of daily life; part III: motor function; part IV: complications). ^*a*^Levodopa equivalent dose was calculated according to Tomlinson et al. (2010).

Global cognitive performance in SDB+ was decreased compared to SDB− (PANDA total score 23.2 ± 3.5 vs. 26.9 ± 2.2, *P* = 0.020, unpaired two-sided *t*-test; Table 5). Also we detected a significantly worse performance in the verbal fluency task of the PANDA in SDB+ patients (*P* = 0.028, Mann–Whitney *U* test; Table 5) attributed to the domains executive function/working memory, attention, semantic memory, and processing speed. Furthermore, in SDB+, an increased amount of missed values in the divided attention tasks assessing cognitive domains executive function and attention was detectable (Table 5). All other domains of TAP and PANDA were similar between both groups.

**TABLE 5:** Cognitive parameters in patient subcohorts with sleep disordered breathing (SDB+, AHI > 5/h) compared to patients without SDB− (AHI ≤ 5/h).

	**SDB+ AHI > 5/h**	**SDB**−**AHI ≤ 5/h**	***P*-values**
**PANDA [*n* = 24]**	**[*n* = 17]**	**[*n* = 7]**	
PANDA (cognition) – overall result	23.2 ± 3.5	26.9 ± 2.2	0.020⁢*§
**Domains memory/executive function**			
Word pair associate immediate recall (raw score)	7.3 ± 2.4	7.7 ± 2.9	0.717^§^
Word pair associate immediate recall (transformed)	4.2 ± 1.0	4.3 ± 1.0	0.951^‡^
Word pair associate delayed recall (raw score)	2.4 ± 1.2	3 ± 1	0.234^‡^
Word pair associate delayed recall (transformed)	4.9 ± 1.6	6 ± 1	0.147^‡^
**Domains executive function (working memory)/semantic memory/attention/processing speed**			
Alternating verbal fluency (raw score)	12.2 ± 3.3	15.1 ± 2.6	0.028*‡
Alternating verbal fluency (transformed)	5.4 ± 1.6	6.4 ± 0.8	0.075^‡^
**Domain visual perception (max. score 3)**			
Visuospatial task (raw score)	2.3 ± 0.9	2.6 ± 0.5	0.710^‡^
Visuospatial task (transformed)	4 ± 1.4	4.6 ± 0.5	0.710^‡^
**Domains executive function/attention (max. score 6)**			
Working memory (raw score)	5.1 ± 0.8	5.6 ± 0.5	0.234^‡^
Working memory (transformed)	4.9 ± 1.2	5.6 ± 0.5	0.234^‡^
Attention task (errors) (raw score)	1.1 ± 2.2	0.1 ± 0.4	0.288^‡^

**TAP [*n* = 14]**	**[*n* = 10]**	**[*n* = 4]**	

**Domains alertness/processing speed**			
Alertness task without auditory cue (median) [ms]	310 ± 63.1 [10]	284.3 ± 12.1	0.244^§^
Alertness task with auditory cue (median) [ms]	307 ± 63.5 [10]	266.8 ± 20.9	0.248^§^
**Domains executive function (response inhibition)/attention**			
Response inhibition (Go/No-Go) task (median) [ms]	473.2 ± 80.7 [9]	479.3 ± 49.3	0.894^§^
Response inhibition (Go/No-Go) task (errors) [*n*]	1.7 ± 1.3 [9]	2.5 ± 2.5	0.710^‡^
**Domains attention/executive function**			
Divided attention task (auditory task) (median) [ms]	775.8 ± 264.5 [9]	642 ± 63.1	0.350^§^
Divided attention task (visual task) (median) [ms]	965.2 ± 197.3 [9]	966.8 ± 127.3	0.989^§^
Divided attention task [errors (*n*)]	5.1 ± 5.6 [9]	3.8 ± 4.3	0.825^‡^
Divided attention task [missed (*n*)]	7.4 ± 5.2 [9]	2 ± 1.4	0.034*‡

Data are median, mean ± SD or number [n] as appropriate. P-values are from ^‡^Mann–Whitney U test or ^§^Student’s unpaired t-test as appropriate. *p < 0.05. PANDA, Parkinson Neuropsychometric Dementia Assessment; SDB, sleep disordered breathing; TAP, Test for Attentional Performance.

### Cognitive Performance in Rapid Eye Movement Sleep Behavior Disorder and Epworth Sleepiness Scale Subgroups

Cognitive measures in patients with abnormal ESS scores indicating daytime sleepiness (ESS > 10) did not differ from patients with normal ESS values (data not shown in detail). When analyzing cognitive performance in patients with (RBD+) and without (RBD−) RBD, no differences were detected in the overall PANDA cognition results and the TAP scores (Table 6), and no relevant differences in clinical and PSG parameters were detected between both groups (Supplementary Table S5).

**TABLE 6:** Cognitive parameters of patient subcohorts with and without REM Sleep Behavior Disorder (RBD).

	**RBD+**	**RBD−**	***P*-values**
**PANDA [*n* = 23]**	**[*n* = 17]**	**[*n* = 6]**	
PANDA (cognition) – overall result	24.9 ± 3.6	22.8 ± 3.7	0.243^§^
**Domains memory/executive function**			
Word pair associate immediate recall (raw score)	8.0 ± 2.4	6.2 ± 2.3	0.125^§^
Word pair associate immediate recall (transformed)	4.5 ± 0.8	3.8 ± 1.2	0.227^‡^
Word pair associate delayed recall (raw score)	2 ± 1.1	2.8 ± 1.1	0.135^‡^
Word pair associate delayed recall (transformed)	5.6 ± 1.4	4.7 ± 1.5	0.201^‡^
**Domains executive function (working memory)/semantic memory/attention/processing speed**			
Alternating verbal fluency (raw score)	12.9 ± 3.8	13.7 ± 1.8	0.609^‡^
Alternating verbal fluency (transformed)	5.5 ± 1.7	6.2 ± 0.8	0.473^‡^
**Domain visual perception**			
Visuospatial task (raw score)	2.5 ± 0.7	2 ± 1.1	0.431^‡^
Visuospatial task (transformed)	4.4 ± 1	3.5 ± 1.6	0.431^‡^
**Domains executive function/attention**			
Working memory (raw score)	5.4 ± 0.8	4.8 ± 0.4	0.135^‡^
Working memory (transformed)	5.2 ± 1.1	4.7 ± 0.8	0.135^‡^
Attention task (errors) (raw score)	0.9 ± 2.2	0.5 ± 0.5	0.473^‡^

**TAP (mean ± *SD*) [*n* = 14]**	**[*n* = 9]**	**[*n* = 5]**	

**Domains alertness/processing speed**			
Alertness task without auditory cue (median) [ms]	299.2 ± 59.9	308.8 ± 48.0	0.765^§^
Alertness task with auditory cue (median) [ms]	284.1 ± 61.7	316.0 ± 46.3	0.336^§^
**Domains executive function (response inhibition)/attention**			
Response inhibition Go/No-Go task (median) [ms]	487.9 ± 77.3 [8]	454.6 ± 59.8	0.431^§^
Response inhibition (Go/No-Go) task (errors) [*n*]	1.9 ± 2 [8]	2 ± 1.4	0.724^‡^
**Domains executive function/attention**			
Divided attention task (auditory task) (median) [ms]	755.1 ± 270.6 [8]	701.8 ± 158.0	0.699^§^
Divided attention task (visual task) (median) [ms]	965.4 ± 184.1 [8]	966.2 ± 175.3	0.994^§^
Divided attention task (errors) (*n*)	4 ± 3.9 [8]	5.8 ± 7.1	0.724^‡^
Divided attention task [missed (*n*)]	6.4 ± 5.4 [8]	4.8 ± 4.8	0.622^‡^

*Data are median, mean ± SD or number [n] as appropriate. P-values are from ^‡^Mann–Whitney U test or ^§^Student’s unpaired t-test as appropriate. PANDA, Parkinson Neuropsychometric Dementia Assessment; RBD, REM sleep behavior disorder; TAP, Test for Attentional Performance.*

### Multiple Linear Regression Analysis

When approaching cognitive performance using a stepwise multiple linear regression model, only the AHI out of all covariates including age, RBD status, sex, education, UPDRS III, and SE showed a significant influence on total PANDA score (Table 3), whereas the association between total PANDA score and age did not survive multiple linear regression analysis. When analyzing the PANDA subtest word pair learning age out of all covariates emerged as a significant factor. In contrast, SE was detected to have a significant influence on the TAP divided attention (visual) task, while missed values in this task were also associated with the AHI (Table 3). Other associations such as between age and the alertness task of the TAP, sex and the Go/No-Go task of the TAP, as well as AHI and the TAP divided attention (auditory) task showed no significant associations in the multiple linear regression analysis (Table 3).

## Discussion

This *post hoc* analysis of the RaSPar study reports detailed analysis of subjective and objective PSG sleep and cognitive measures in a cohort of 26 PD patients with sleep disturbances. Our study population consists of patients with sleep disturbances with mild to moderate disease severity. Most frequent subjective sleep complaints were SMI, daytime sleepiness, RBD, and SOI. In line with our results, one of the most frequent sleep complaints in PD in previous studies is insomnia with a prevalence of up to 90% (Zoccolella et al., 2011). PSG data underlined the subjective perception of sleep disturbances in our PD patient cohort showing impairment of sleep quality such as reduced SE and REM sleep proportion. Additionally, mild to moderate SDB was detected in a high proportion of patients, while severe SDB was an exclusion criterion for study participation. The frequency of EDS in our cohort (89%) is higher than reported in epidemiological studies with 20–50% of patients suffering from EDS (Gjerstad et al., 2006), but is in line with other studies with up to 87% of patients affected (Kurtis et al., 2013).

Apart from the fact that all patients had a total PANDA score within the normal range, slight cognitive impairment in several subdomains was detectable and both subjective and objective sleep disturbances were shown to be associated with cognitive performance. When comparing results of our cohort with normative values of the TAP, impairment in nearly all tasks with emphasis on the attentional domain as well as alertness compared to executive function/response inhibition was demonstrated. In line with our results, impairment of attention and processing speed, executive function/working memory, and semantic memory is most prominent in PD patients (Weintraub et al., 2015). Dujardin et al. (2013) detected alternating attention deficits in PD patients compared to healthy controls but no impairment in attention and divided attention tasks. These differences might be explained by the influence of sleep disturbances in our cohort. Our findings are in line with basic science results in a zebrafish model of PD, where sleep deprivation led to exacerbation of short-term cognitive deficits and dysphoria (Lv et al., 2019) and reports from clinical settings demonstrating impaired global cognitive performance in PD patients with questionnaire questionnaire-assessed sleep disturbances (Junho et al., 2018).

Accordingly, impairment of PSG-measured sleep quality, e.g., shown by reduced SE and TST, was associated with impaired cognitive performance, particularly affecting cognitive domains attention and divided attention in the PANDA and TAP. Accordingly, other sleep quality parameters such as the AI and percentage of REM sleep were associated with the cognitive domains alertness, attention as well as semantic and working memory. This is in line with other studies showing impairment of memory function by disturbed sleep either by decreased percentage of REM sleep or by decreased SE (Scullin and Bliwise, 2015) and also negative effects of sleep fragmentation on executive function in PD (Stavitsky et al., 2012), though again this study did not include PSG measurements of sleep.

RBD in PD patients and the akinetic PD subtype were found to be associated with impaired cognitive function in many studies (Svenningsson et al., 2012; Jozwiak et al., 2017; Pilotto et al., 2019). Accordingly, overall cognitive performance particularly affecting visuospatial function, executive function, attention, and memory in PD patients with RBD compared to those without was demonstrated (Huang et al., 2018). In contrast to these reports, we were not able to detect an association between PD subtypes as well as the presence of RBD with distinct cognitive measures. However, some of the other studies did not rely on PSG but on patients’ and spouses’ or caregivers’ perspective to diagnose RBD. This is of great importance as this leads to selection bias with only violent RBD being diagnosed as such but not mild forms of RBD as already known (Sixel-Doring et al., 2016). This and also the relatively small cohort included in our study might explain these discrepancies.

The prevalence of SDB was elevated in our preselected cohort of patients with sleep disturbances. SDB was shown to impair cognitive performance with negative influences on attention, semantic memory, and executive function/working memory. These are promising results to further evaluate cognitive impairment caused by SDB as we excluded patients with severe SDB, which might have even strengthened our results. In line with our results, Huang et al. (2018) showed impaired cognitive performance in PD patients with SDB, especially in delayed recall tasks assessing the domains semantic and working memory in particular. The presence of RBD alleviated OSA severity, but combination of OSA and RBD exacerbated cognitive deficits (Huang et al., 2018). In contrast to our findings, Scullin and colleagues were not able to detect an influence of AHI on cognition. This might be due to selection bias of patients with only very low AHI of 7 ± 3.2/h in this population, which is not representative for SDB patients (Scullin et al., 2015). In line with others (De Cock et al., 2008; Trotti and Bliwise, 2010), we did not detect an association between SDB and BMI, underlining the assumption that the pathophysiology of SDB seems to differ between PD patients and the general population, which means that the clinical features pointing toward SDB in the general population seem not to be helpful in identifying patients at risk in the PD population (Trotti and Bliwise, 2010).

Interestingly, also subjective sleep complaints such as EDS and insomnia were associated with impairment in the corresponding cognitive tasks, particularly interfering with attention, executive function, and to a lesser extent memory. This is of high importance for clinical practice, emphasizing the need to thoroughly evaluate sleep disturbances in PD as sleep seems to influence cognitive performance and quality of life to a relevant extent. Sleep issues as well as comorbidities such as depression should be addressed regularly in routine care, which underlines the need for adequate diagnostic and therapeutic tools to address these issues. Complementary, screening for sleep apnea in PD patients, particularly in those with EDS or symptoms of insomnia, should be applied generously to detect SDB as this is mandatory to treat sleep disturbances and might be effective in preventing further cognitive decline and even motor progression (Kaminska et al., 2018; Meng et al., 2019). As SDB is a treatable cause for sleep disturbances, patients should be evaluated regarding sleep disturbances more consequently, and treatment options such as positive airway pressure therapy should be applied in these patients. Treatment options for insomnia including cognitive behavioral therapy and consequent treatment of comorbid depression should be integrated into routine care of PD patients, and special care programs should be implemented to prevent sleep disturbance-related cognitive decline and impairment of quality of life of PD patients and caregivers. Given the fact that research in this area, particularly randomized multicenter studies with pharmacological substances targeting sleep disturbances, is scarce, our results underline the urge to enhance efforts in this field.

Limitations of this work comprise the relatively small sample size, only enabling a relatively small number of statistical sub-analyses, thus lacking statistical power to control for all potential demographic and other variables, which might influence our results. Also, our work comprises only cross-sectional data. Another limitation might be that cognitive testing was performed before PSG recording, thus we cannot rule out discrepancies between sleep and cognitive performance. On the other hand, first-night effects of PSG monitoring can be ruled out in explaining cognitive performance. These limitations notwithstanding, we were able to detect relevant influences of subjective as well as objective sleep disturbances, such as SDB and impaired sleep quality, measured by the gold standard PSG, on cognitive functions in PD patients. However, we were able to identify SDB, age, and SE as important factors for explaining variance in cognitive dysfunction by multivariate regression analysis.

In conclusion, patients with daytime sleepiness or cognitive impairment should be thoroughly evaluated regarding sleep disturbances and especially SDB as a treatable cause for cognitive impairment. However, larger PD populations should be analyzed to further elucidate mechanisms of the interaction between sleep and cognition, and multicenter intervention studies on sleep disturbances should be implemented to evaluate the long-term outcome of cognitive impairment with and without interventions.

## Data Availability Statement

All datasets generated for this study are included in the article/Supplementary Material.

## Ethics Statement

The current work is a *post hoc* analysis of a single-center, double-blind, baseline-controlled clinical RaSPar trial, which was conducted by Technische Universität Dresden, Germany (EudraCT-no.: 2010-023756-8) according to the ethical principles of the Declaration of Helsinki and the German drug law (12th version, AMG) and was registered with ClinicalTrials.gov (NCT01442610) (Schrempf et al., 2018). It was approved by the local institutional review board (Ethics Committee of the Technische Universität Dresden; EK161052011) and the German Federal Institute for Drugs and Medical Devices (BfArM). Written informed consent was obtained from all study participants prior to study inclusion. An independent CRO (KKS Dresden, TU Dresden, Germany) was responsible for data management and monitoring.

## Author Contributions

WH contributed to the design and conceptualization of the study, analysis and interpretation of data, and drafting and revising the manuscript for intellectual content. HS-P, MF, MW, CF, KO, MB, and ML contributed to the provision of study material and revising the manuscript for intellectual content. EK contributed to the analysis and interpretation of data and revising the manuscript for intellectual content. HR contributed to revising the manuscript for intellectual content. AS contributed to the design and conceptualization of the study and drafting and revising the manuscript for intellectual content. All authors contributed to the article and approved the submitted version.

## Conflict of Interest

WH has received an unrestricted research grant from TEVA GmbH, Berlin, Germany, during the conduct of the study. MF reports no disclosures related to the study but received an unrestricted research grant from Deutsche Forschungsgemeinschaft (German Research Council). ML reports no disclosures during the conduct of the study but received honoraria for presentations/advisory boards from UCB Pharma, Grünenthal, and Abbvie. HR has received grants and honoraria from TEVA and Lundbeck GmbH during the conduct of the study and was acting on advisory boards and gave lectures and received research grants from Abbott AbbVie, Bayer Health Care, Bial, Boehringer Ingelheim, Brittania, Cephalon, Desitin, GSK, Lundbeck, Medtronic, Merck-Serono, Novartis, Orion, Pfizer, TEVA, UCB Pharma, Valeant, and Zambon outside the submitted work. AS has received an unrestricted research grant from TEVA GmbH, Berlin, Germany, during the conduct of the study and has received funding from the Bundesministerium für Wirtschaft und Technologie (Federal Ministry of Economic Affairs and Technology), the Deutsche Forschungsgemeinschaft (German Research Association), the Helmholtz Association, the NCL Foundation, and the Novartis Foundation. He has received unrestricted research grants from TEVA Pharma and Global Kinetics Corporation (GKC, Melbourne, Australia) and honoraria for presentations/advisory boards/consultations from Desitin, Abbvie, GKC, Mundipharma, Zambon, Pfizer, Lund University, and Volkswagen Foundation. He has received royalties from Kohlhammer Verlag and Elsevier Press. He serves as an editorial board member of Stem Cells, Stem Cells International, Open Biotechnology Journals, and jbc The Journal of Biological Chemistry outside the submitted work.

The remaining authors declare that the research was conducted in the absence of any commercial or financial relationships that could be construed as a potential conflict of interest.
