# Supplementary material for: Sleep Disturbances and Sleep Disordered Breathing Impair Cognitive Performance in Parkinson’s Disease
Source: Front Neurosci. 2020 Aug 6;14:689. doi: 10.3389/fnins.2020.00689 (PMC7438827; doi:10.3389/fnins.2020.00689)
Supplement: Supplementary file 1 [file Table_1.pdf]

Supplementary Table S1. Polysomnographic sleep measures of the total cohort

	Total cohort (n=29)
**Polysomnographic sleep parameters**	
Sleep efficiency (TST/TIB, %)	64.5±18.4
Sleep maintenance (TST/SPT, %)	70.5±15
Total sleep time (TST, min)	318.1±93.9
Stage N1 (%)	17±9
Stage N2 (%)	49.6±10.2
Stage N3 (slow wave sleep %)	22.2±15.5
Stage REM (%)	11.2±7
Sleep latency (min)	25.5±46.6
REM-sleep latency (min)	172±92.1
Wake (in TIB) (min)	175.7±93.8
Arousal-Index (n/h)	44.8±12.8
Apnoea-Hypopnoea-Index (AHI; n/h)	10.9±8.5
Respiratory-Distress-Index (RDI; n/h)	19.3±10.5
AHI ≤ 5/h = no SDB	7/26 (27%)
AHI > 5 < 15/h = mild SDB	12/26 (46%)
AHI >15 < 30/h = moderate SDB	7/26 (27%)
Oxygen-Desaturation-Index (ODI; n/h)	6.2±6
ODI NREM (n/h)	5.9±5.7
ODI REM (n/h)	7.8±14.0
PLM-Index (n/h)	16.3±27.4
PLM-Arousal-Index (n/h)	2.8±4.1
PLM-Index ≤ 5/h	14/26 (54%)
PLM-Index > 5/h	4/26 (15%)
PLM-Index > 15/h	8/26 (31%)
REM Sleep Behavior Disorder in PSG	18/25 (69%)

Data are mean ± standard deviation (SD) or percentages (%).

AHI=Apnoe-Hypopnoe-Index; ODI=Oxygen Desaturation Index; PLM=Periodic limb movements; PSG=polysomnography; REM=Rapid eye movement sleep; SPT=Sleep partial time; SDB=sleep disordered breathing; stage N1 and N2=light sleep stages, stage N3=slow wave sleep; TIB=Time in bed; TST=Total sleep time
