# Supplementary material for: Sleep Disturbances and Sleep Disordered Breathing Impair Cognitive Performance in Parkinson’s Disease
Source: Front Neurosci. 2020 Aug 6;14:689. doi: 10.3389/fnins.2020.00689 (PMC7438827; doi:10.3389/fnins.2020.00689)
Supplement: Supplementary file 2 [file Table_2.pdf]

Supplementary Table S2. Correlations between cognitive performance, clinical and polysomnographic sleep parameters

<table>
<tr><th></th><th>age</th><th>H&Y</th><th>UPDRS II</th><th>UPDRS IV</th><th>PSQI</th><th>SE (TST/TIB)</th><th>SMI (TST/SPT)</th><th>TST</th><th>AI (n/h)</th><th>SL (min)</th><th>REM (%)</th></tr>
<tr><td>PANDA total score</td><td>|r|=-0.443
P=0.030*</td><td>n.s.</td><td>n.s.</td><td>n.s.</td><td>n.s.</td><td>n.s.</td><td>n.s.</td><td>n.s.</td><td>n.s.</td><td>n.s.</td><td>n.s.</td></tr>
<tr><td>PANDA word pair – immediate recall</td><td>|r|=-0.424
P=0.039*</td><td>n.s.</td><td>n.s.</td><td>n.s.</td><td>|r|=-0.451
P=0.027*</td><td>n.s.</td><td>n.s.</td><td>n.s.</td><td>n.s.</td><td>n.s.</td><td>n.s.</td></tr>
<tr><td>PANDA word pair – delayed recall</td><td>|r|=-0.408
P=0.048*</td><td>n.s.</td><td>n.s.</td><td>n.s.</td><td>n.s.</td><td>n.s.</td><td>n.s.</td><td>n.s.</td><td>n.s.</td><td>n.s.</td><td>|r|=0.426
P=0.038*</td></tr>
<tr><td>PANDA attention (errors, [n])</td><td>n.s.</td><td>n.s.</td><td>n.s.</td><td>n.s.</td><td>|r|=0.407
P=0.049*</td><td>n.s.</td><td>|r|=-0.436
P=0.033*</td><td>n.s.</td><td>n.s.</td><td>n.s.</td><td>n.s.</td></tr>
<tr><td>PANDA verbal fluency</td><td></td><td></td><td></td><td></td><td>|r|=-0.452
P=0.027*</td><td></td><td></td><td></td><td></td><td></td><td></td></tr>
<tr><td>TAP- Alertness w/o aud. cue – (median, [msec])</td><td>n.s.</td><td>n.s.</td><td>n.s.</td><td>n.s.</td><td>n.s.</td><td>n.s.</td><td>n.s.</td><td>n.s.</td><td>|r|=0.548
P=0.043*</td><td>n.s.</td><td>n.s.</td></tr>
<tr><td>TAP- Alertness w.aud. cue – (median, [msec])</td><td>n.s.</td><td>n.s.</td><td>|r|=0.564
P=0.036*</td><td>n.s.</td><td>n.s.</td><td>n.s.</td><td>n.s.</td><td>n.s.</td><td>n.s.</td><td>n.s.</td><td>n.s.</td></tr>
<tr><td>TAP – Go/No-Go – (median, [msec])</td><td>n.s.</td><td>|r|=0.668
P=0.013*</td><td>n.s.</td><td>n.s.</td><td>n.s.</td><td>n.s.</td><td>n.s.</td><td>n.s.</td><td>n.s.</td><td>n.s.</td><td>n.s.</td></tr>
<tr><td>TAP – Go/No-Go – (errors, [n])</td><td>n.s.</td><td>n.s.</td><td>n.s.</td><td>|r|=-0.643
P=0.018*</td><td>n.s.</td><td>n.s.</td><td>n.s.</td><td>n.s.</td><td>n.s.</td><td>n.s.</td><td>n.s.</td></tr>
<tr><td>TAP – divided attention – aud. (median, [msec])</td><td>n.s.</td><td>n.s.</td><td>n.s.</td><td>n.s.</td><td>n.s.</td><td>n.s.</td><td>n.s.</td><td>n.s.</td><td>n.s.</td><td>n.s.</td><td>|r|=-0.571
P=0.042*</td></tr>
<tr><td>TAP – divided attention – vis. (median, [msec])</td><td>n.s.</td><td>n.s.</td><td>|r|=0.592
P=0.033*</td><td>n.s.</td><td></td><td>|r|=-0.640
P=0.018*</td><td>|r|=-0.585
P=0.036*</td><td>|r|=-0.739
P=0.004*</td><td>n.s.</td><td>n.s.</td><td>n.s.</td></tr>
<tr><td>TAP – divided attention (errors, [n])</td><td>n.s.</td><td>n.s.</td><td>|r|=0.557
P=0.048*</td><td>n.s.</td><td>n.s.</td><td>n.s.</td><td>n.s.</td><td>n.s.</td><td>n.s.</td><td>|r|=0.699
P=0.008*</td><td>n.s.</td></tr>
<tr><td>TAP – divided attention (missed, [n])</td><td>n.s.</td><td>n.s.</td><td>n.s.</td><td>n.s.</td><td>n.s.</td><td>n.s.</td><td>n.s.</td><td>n.s.</td><td>n.s.</td><td>n.s.</td><td>|r|=-0.612
P=0.026*</td></tr>
</table>

Results are displayed as results of correlation tests (Pearson correlation test or Spearman correlation test) as appropriate. Non-significant tasks and results (e.g., TAP alertness task without cueing / Go/No-Go) were omitted for clarity. κ or Pearson's correlation coefficient $|r|<0.3$ was considered a weak, κ/|r|=0.3–0.59 a moderate, $\kappa/|r|\geq0.6$ a strong agreement/correlation. Bold values represent significant results.

AI=Arousal Index; H&Y=Hoehn & Yahr stage; Go/No-Go=response inhibition task within the TAP; n.s.=non-significant; PANDA=Parkinson Neuropsychometric Dementia Assessment; PD=Parkinson's disease; PDQ-39=Parkinson's Disease Questionnaire; PDSS-2=Parkinson's Disease Sleepiness Scale 2; PSQI=Pittsburgh Sleep Quality Index; REM=Rapid eye movement sleep; SE=Sleep efficiency; SL=Sleep latency; SMI=Sleep maintenance; SPT=Sleep period time; TAP: Test of Attentional Performance; TIB=Time in bed; TST=Total sleep time; UPDRS=Unified Parkinson's disease rating scale (part I: evaluation of mentation, behavior and mood; part II: activities of daily life; part III: motor function; part IV: complications).
