# Supplementary material for: Sleep Disturbances and Sleep Disordered Breathing Impair Cognitive Performance in Parkinson’s Disease
Source: Front Neurosci. 2020 Aug 6;14:689. doi: 10.3389/fnins.2020.00689 (PMC7438827; doi:10.3389/fnins.2020.00689)
Supplement: Supplementary file 3 [file Table_3.pdf]

Supplementary Table S3. Correlations between cognitive performance and parameters of sleep disordered breathing

	AHI (n/h)	RDI (n/h)	ODI (n/h)	RDI / REM (n/h)	RDI / NREM (n/h)	Mean duration of apnea / hypopnea	Mean SpO2 in NREM (%)	MeanSpO2 in wake (%)	SpO2 <90%	Hypopnea-Index (n/h)
**PANDA – total score**	**|r|=-0.557, *P*=0.005***	**|r|=-0.501, *P*=0.013***	**|r|=-0.499 *P*=0.013***	n.s.	**|r|=-0.457 *P*=0.025***	n.s.	n.s.	n.s.	n.s.	n.s.
**PANDA word pair – immediate**	n.s.	n.s.	n.s.	n.s.	n.s.	**|r|=0.437 *P*=0.033***	**|r|=0.492 *P*=0.015***	n.s.	n.s.	n.s.
**PANDA word pair – delayed**	n.s.	n.s.	n.s.	n.s.	n.s.	**|r|=0.533 *P*=0.007***	**|r|=0.473 *P*=0.020***	**|r|=0.457 *P*=0.025***	n.s.	n.s.
**PANDA – working memory**	n.s.	n.s.	n.s.	n.s.	n.s.	**|r|=0.427 *P*=0.038***	n.s.	n.s.	**|r|=-0.519 *P*=0.009***	n.s.
**PANDA attention / errors**	n.s.	n.s.	n.s.	**n.s.**	n.s.	n.s.	n.s.	n.s.	n.s.	n.s.
**TAP- alertness with aud. cue –** (median, [msec])	n.s.	n.s.	n.s.	n.s.	n.s.	n.s.	n.s.	n.s.	n.s.	**|r|=0.675 *P*=0.008***
**TAP – divided attention – aud.** (median, [msec])	n.s.	**|r|=0.714 *P*=0.006***	n.s.	n.s.	**|r|=0.709 *P*=0.007***	n.s.	n.s.	n.s.	n.s.	n.s.
**TAP – divided attention – vis.** (median, [msec])	n.s.	n.s.	n.s.	n.s.	n.s.	n.s.	n.s.	n.s.	n.s.	n.s.
**TAP – divided attention** (missed, [n])	**|r|=0.558 *P*=0.048***	n.s.	n.s.	n.s.	n.s.	n.s.	n.s.	n.s.	n.s.	n.s.

Results are displayed as results of correlation tests (Pearson correlation test or Spearman correlation test) as appropriate. Non-significant tasks and results (e.g., TAP alertness task without cueing / Go/No-Go) were omitted for clarity. κ or Pearson's correlation coefficient |r|<0.3 was considered a weak, κ/|r|=0.3–0.59 a moderate, κ/|r|≥0.6 a strong agreement/correlation. Bold values represent significant results.

AHI=Apnoe-Hypopnoe-Index; ODI=Oxygen Desaturation; PANDA= PANDA=Parkinson Neuropsychometric Dementia Assessment; RDI=Respiratory Disturbance Index; REM=Rapid Eye Movements; SpO2=Oxygen Saturation; SPT=Sleep partial time; SDB=sleep disordered breathing; stage N1 and N2=light sleep stages, stage N3=slow wave sleep; NREM= nicht-REM-assoziierter Schlaf; TAP=Test for Attentional Performance; TIB=Time in bed; TST=Total sleep time
