# Supplementary material for: Sleep Disturbances and Sleep Disordered Breathing Impair Cognitive Performance in Parkinson’s Disease
Source: Front Neurosci. 2020 Aug 6;14:689. doi: 10.3389/fnins.2020.00689 (PMC7438827; doi:10.3389/fnins.2020.00689)
Supplement: Supplementary file 4 [file Table_4.pdf]

Supplementary Table S4. Polysomnographic sleep characteristics of patient subcohorts with Sleep disordered breathing (SDB+, AHI > 5/h) and without SDB (SDB-, AHI ≤ 5/h)

	SDB+ AHI > 5/h [n=19]	SDB- AHI ≤ 5/h [n=7]	*P* value
**Polysomnographic measures (mean±SD)**			
Sleep efficiency (TST/TIB in %)	61.4±19.6	73±11.7	0.159§
Sleep maintenance (TST/SPT in %)	67.9±15.3	77.4±12.9	0.159§
Total sleep time (TST, min)	304.9±103	353.9±53.7	0.247§
Wake-time in TIB (min)	191.4±100.7	133.1±58	0.188‡
Sleep stage N1 (%)	17.9±9.4	14.6±7.9	0.421§
Sleep stage N2 (%)	49.6±11.6	49.6±5.3	0.984§
Sleep stage N3 (%)	22.8±17.4	20.5±9.4	0.744§
Sleep stage REM (%)	9.7±6.8	15.2±6.2	0.074§
Sleep latency (min)	30.1 ±53.9	12.8±4.9	1.000‡
REM Sleep latency (min)	166.7±90.7	184.1±101.4	0.720‡
Arousal Index (n/h)	47.1±12.4	38.5±12.4	0.131§
Apnoea-Hypopnea-Index (n/h)	14.2±7.4	1.8±1.5	**<0.001§ ****
Respiratory Distress Index (n/h)	23.5±8.6	8±6	**<0.001§ ****
ODI(n/h)	8.3±5.7	0.6±0.9	**<0.001‡ ****
ODI (n/h) NREM	7.9±5.4	0.5±0.9	**<0.001‡ ****
ODI (n/h) REM	10.9±15.9	0.8±1.1	**0.033‡ ***
PLM index (n/h)	20.7±30.8	4.3±6.9	0.107‡
PLM-Arousal-Index (n/h)	3.6±4.5	0.8±1.8	**0.047‡ ***
REM sleep behavior disorder	12/18 (67%)	6/7 (85%)	0.626‡‡‡‡

Data are mean±SD or numbers (%) as appropriate. *P* values are from §student's t-test, ‡‡‡‡Fishers exact test or ‡Mann-Whitney-U-test as appropriate. **P* < 0.05, ***P* < 0.01, bold values represent significant results.

AHI=Apnoe-Hypopnoe-Index; ODI=Oxygen Desaturation Index; PLM=Periodic limb movements; PSG=polysomnography; REM=Rapid eye movement sleep; SPT=Sleep partial time; SDB=sleep disordered breathing; stage N1 and N2=light sleep stages, stage N3=slow wave sleep; TIB=Time in bed; TST=Total sleep time
