# Supplementary material for: Sleep Disturbances and Sleep Disordered Breathing Impair Cognitive Performance in Parkinson’s Disease
Source: Front Neurosci. 2020 Aug 6;14:689. doi: 10.3389/fnins.2020.00689 (PMC7438827; doi:10.3389/fnins.2020.00689)
Supplement: Supplementary file 5 [file Table_5.pdf]

Supplementary Table S5. Clinical and polysomnographic data of patients with (RBD+) and without REM sleep behavior disorder (RBD-)

	RBD+ [n=18]	RBD- [n=7]	*P*-values
**Clinical and Demographic characteristics**			
Age (years)	70±6.8 (56 – 78)	73±4.6 (66 – 80)	0.289[§]
Men/women (n)	9 m; 9 f	6 m, 1 f	0.179[#]
PD symptom duration (years)	4.6±3.8 (0 – 15)	2.4±3.3 (0 - 8)	0.110[‡]
PD duration (years)	2.8±2.8 (0 – 10)	2.1±3.4 (0 - 8)	0.389[‡]
UPDRS total score	32.4±11.8	30.9±11.3	0.763[§]
UPDRS part I (psychiatric)	2.7±1.4	2.1±1.5	0.423[‡]
UPDRS part II	8.4±3.8	8.7±3.1	0.870[§]
UPDRS part III (motor function)	18.3±7.4	18.3 ± 7.9	0.989[‡]
UPDRS part IV (motor complications)	2.9±2.0	1.7±1.5	0.085[‡]
Modified Hoehn & Yahr stage	2±0.8	2.1±0.6	0.976[‡]
1	6/18 (30%)	1/7 (14%)	0.396[#]
2	4/18 (22%)	4/7 (57%)	
2.5	3/18 (17%)	1/7 (14%)	
3	5/18 (28%)	1/7 (14%)	
Schwab & England Scale ADL	85±8.6	90.0±8.9 [6]	0.343[‡]
PDQ-39	42.9±29.5 [17]	29.4±23.2	0.294[§]
PDSS-2	21.5±7.8 [17]	15.7±9.1	0.129[§]
ESS	9.4±5.6 [17]	7.3±3.4	0.379[§]
ESS < 10	10/17 (59%)	6/7 (86%)	0.352[‡‡]
ESS > 10	7/17 (41%)	1/7 (14%)	0.352[‡‡]
PSQI	10.3±2.9	8.6±2	0.154[§]
MADRS	9.8±6.5	7.9±6.6	0.495[‡]
PANDA (mood)	3.3±2.3	2.5±2.6	0.513[§]
BMI (kg/m^2)	26.1±3.8 (17 - 33)	28.3±4.1 (24 - 35)	0.226[§]
Levodopa equivalent dose (mg/day)	339.9±230.3 (0 – 860)	245±233.4 (0 – 700)	0.366[§]
**Polysomnographic Sleep Parameters** (mean±SD)			
Sleep efficiency (TST/TIB in %)	68.4±17.3	61.3±11.8	0.331[§]
Sleep maintenance (TST/SPT in %)	74.6±12.8	65.3±12.2	0.113[§]
Total sleep time (TST, min)	337.6±92.3	299.9±53	0.323[§]
Wake-time in TIB (min)	154.1±79.6	191.7±65.8	0.141[‡]
Sleep stage N1 (%)	16.1±9.6	19.6±7.9	0.392[§]
Sleep stage N2 (%)	50.7±11	48.3±8	0.618[§]
Sleep stage N3 (%)	21±17.2	21.9±8.7	0.900[§]
Sleep stage REM (%)	12.3±7.2	10.2±5.4	0.496[§]
Sleep latency (min)	18.5±21.4	13.7±15.8	0.270[‡]
REM Sleep latency (min)	198±92.8	98.3±31.4	**0.006**[‡] **
Arousal Index (n/h)	45.8±13.4	41±11.8	0.416[§]
AHI (n/h)	10.2±8.5	12.7±9.5	0.524[§]
RDI (n/h)	18.4±10.8	21.3±11.1	0.560[§]
ODI (n/h)	5±5.2	8.8±7.6	0.178[‡]
ODI NREM n/h)	5.1±5.4	7.6±6.6	0.297[‡]
ODI REM (n/h)	3.6±6.3	19.9±22.3	0.016[‡]
Total PLM index (n/h)	15.9±27.6	19.1±30.6	0.615[‡]
PLM-Arousal-Index (n/h)	2.9±4.1	2.8±4.6	0.852[‡]

Data are mean±SD, range (min – max), numbers [n] or percentages (%) as appropriate. *P* values are from [§]student's t-test, [#]Fisher's exact test or [‡]Mann-Whitney-U-test as appropriate. $^{**}P < 0.01$, bold values represent significant results.

ADL=Activities of daily living; AHI=Apnea-hypopnea index; BMI=Body-Mass-Index; ESS=Epworth sleepiness scale; LED=Levodopa equivalent dose; MADRS=Montgomery Asberg Depression Rating Scale; PANDA=Parkinson Neuropsychometric Dementia Assessment; PD=Parkinson's disease; PDQ-39=Parkinson's Disease Questionnaire; PDSS-2=Parkinson's Disease Sleepiness Scale 2; PLM=Periodic limb movements; PSQI=Pittsburgh Sleep Quality Index; RDI=respiratory distress index (sleep-associated events including respiratory effort related arousals); REM=REM Sleep; SD=Standard deviation; SPT=Sleep period time; TIB=Time in bed; TST=Total sleep time. UPDRS=Unified Parkinson's disease rating scale (part I: evaluation of mentation, behavior and mood; part II: activities of daily life; part III: motor function; part IV: complications).

[a]Levodopa equivalent dose was calculated according to Tomlinson and co-workers [55].
