# Supplementary material for: Sleep Disturbances and Sleep Disordered Breathing Impair Cognitive Performance in Parkinson’s Disease
Source: Front Neurosci. 2020 Aug 6;14:689. doi: 10.3389/fnins.2020.00689 (PMC7438827; doi:10.3389/fnins.2020.00689)
Supplement: Supplementary file 6 [file Image_1.pdf]

Figure 1. Cognitive performance in patients with and without sleep disordered breathing (SDB)

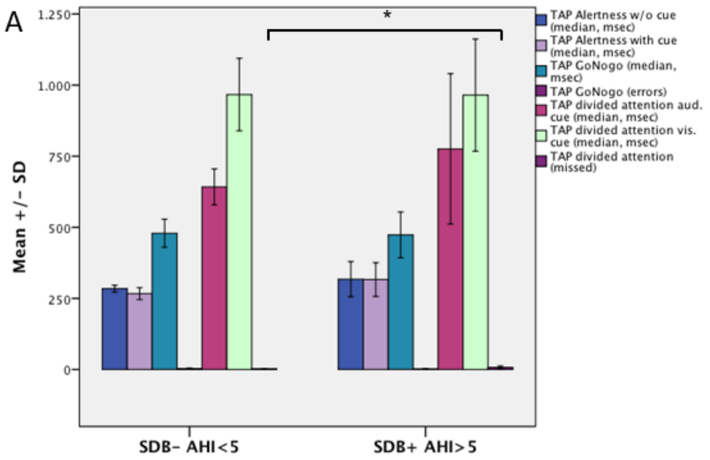

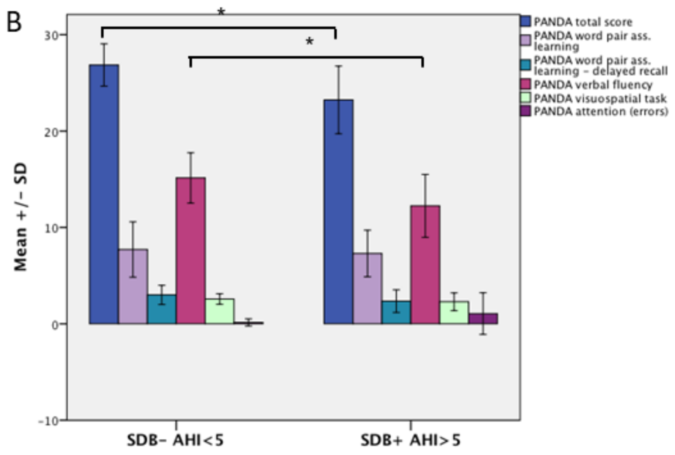

Figure 1A: Test of Attentional Performance comparing different tasks/domains in PD patients without sleep disordered breathing (SDB-, AHI ≤ 5/h) compared to patients with SDB (SDB+, AHI > 5/h)

*p<0.05, AHI=Apnoe-Hypopnoe-Index

Figure 1B: Parkinson Neuropsychometric Dementia Assessment (raw data) comparing different tasks in PD patients without sleep disordered breathing (SDB-, AHI ≤ 5/h) compared to patients with SDB (SDB+, AHI > 5/h)

*p<0.05, AHI=Apnoe-Hypopnoe-Index
